# MANF ameliorates DSS-induced mouse colitis via restricting Ly6C^hi^CX3CR1^int^ macrophage transformation and suppressing CHOP-BATF2 signaling pathway

**DOI:** 10.1038/s41401-022-01045-8

**Published:** 2023-01-12

**Authors:** Lin Yang, Wen-wen Shen, Wei Shao, Qing Zhao, Gao-zong Pang, Yi Yang, Xiao-fang Tao, Wei-ping Zhang, Qiong Mei, Yu-xian Shen

**Affiliations:** 1 School of Basic Medical Sciences, Anhui Medical University, Hefei, 230032 China; 2 Biopharmaceutical Institute, Anhui Medical University, Hefei, 230032 China; 3 First Affiliated Hospital of Anhui Medical University, Hefei, 230032 China

**Keywords:** inflammatory bowel disease, colitis, MANF, macrophages, Th17 cells, BATF2-mediated innate immune response, CHOP-BATF2 signaling

## Abstract

Mesencephalic astrocyte-derived neurotrophic factor (MANF), an endoplasmic reticulum stress-inducible secreting protein, has evolutionarily conserved immune-regulatory function that contributes to the negative regulation of inflammation in macrophages. In this study, we investigated the profiles of MANF in the macrophages of the patients with active inflammatory bowel disease (IBD) and the mice with experimental colitis, which was induced in both myeloid cell-specific MANF knockout mice and wild-type mice by 3% dextran sodium sulfate (DSS) for 7 days. We found that MANF expression was significantly increased in intestinal macrophages from both the mice with experimental colitis and patients with active IBD. DSS-induced colitis was exacerbated in myeloid cell-specific MANF knockout mice. Injection of recombinant human MANF (rhMANF, 10 mg·kg^–1^·d^–1^, i.v.) from D4 to D6 significantly ameliorated experimental colitis in DSS-treated mice. More importantly, MANF deficiency in myeloid cells resulted in a dramatic increase in the number of Ly6C^hi^CX3CR^int^ proinflammatory macrophages in colon lamina propria of DSS-treated mice, and the proinflammatory cytokines and chemokines were upregulated as well. Meanwhile, we demonstrated that MANF attenuated Th17-mediated immunopathology by inhibiting BATF2-mediated innate immune response and downregulating CXCL9, CXCL10, CXCL11 and IL-12p40; MANF functioned as a negative regulator in inflammatory macrophages via inhibiting CHOP-BATF2 signaling pathway, thereby protecting against DSS-induced mouse colitis. These results suggest that MANF ameliorates colon injury by negatively regulating inflammatory macrophage transformation, which shed light on a potential therapeutic target for IBD.

## Introduction

Inflammatory bowel disease (IBD) is characterized by chronic relapsing inflammation of the gastrointestinal (GI) tract. The two major categories of IBD are Crohn’s disease (CD) and ulcerative colitis (UC) [1]. Inflammation in CD is characterized by patchy, transmural inflammation that can occur anywhere in the alimentary tract, whereas UC presents as continuous, more superficial inflammation of the mucosa and submucosa that is limited to the colon [2]. The incidence of IBD is increasing worldwide, affecting the health of over 1 million residents in the USA and 2.5 million in Europe, especially in newly industrialized countries (i.e., Asia, South America and Middle East) [3]. However, current treatments can only maintain remission in some individuals but cannot completely control inflammation and recurrence. The emerging evidence showed that chronic inflammatory status of IBD leads to a high risk of colitis-related cancer. According to statistics, 20% of IBD patients will develop colitis-related cancer, and the mortality rate can exceed 50% [4].

The pathogenesis of IBD is complex and largely unknown. It mainly involves four aspects: genetic, environmental, microbial and immune response disorders [5]. Immunologically, IBD is currently thought to be due to the disruption of multiple regulatory mechanisms that maintain intestinal homeostasis, resulting in an abnormal innate and/or adaptive immune response to the intrinsic gut flora in genetically susceptible individuals [6]. In the past few decades, most studies on IBD have investigated abnormal adaptive immunity [7]. However, in recent years, the focus has shifted toward mucosal innate immunity, and the central role of macrophages in the pathogenesis of IBD has been highlighted [8, 9].

The intestinal immune system is the largest and most complex component of the immune system in humans. It is continuously exposed to a range of foreign antigens and must distinguish between harmful and harmless antigens to ensure an appropriate response. On the one hand, it must maintain tolerance to a vast array of antigens derived from food and dense but largely harmless commensal bacteria. On the other hand, it must be ever ready to respond to potentially life-threatening pathogens that aim to insult via the oral route. Failure to achieve this balance between tolerance and reactivity can lead to the development of chronic inflammatory diseases such as IBD [10]. The innate immune response induced by intestinal macrophages through their surface and cytosolic expression of pathogen-associated molecular patterns serves as the first line of defense against invading pathogens [5]. In the steady state, CX3CR1^hi^ (CX3CR1 high)-resident macrophages maintain intestinal homeostasis and protect the host from certain pathogens by engulfing apoptotic cells, promoting the development of regulatory T cells, or supporting the proliferation of intestinal epithelial progenitors [11]. However, in inflammatory conditions, Ly6C^hi^ (Ly6C high) monocytes from the peripheral circulating blood infiltrate massively into the intestinal mucosa and differentiate into plastic CX3CR1^int^ (CX3CR1 intermediate positive) proinflammatory macrophages. They express higher levels of proinflammatory mediators and Toll-like receptors (TLRs), nitric oxide, reactive oxygen intermediates, and metalloproteinases compared to CX3CR1^hi^-resident macrophages that dominate in the healthy colon. In the pathogenesis of IBD, Ly6C^hi^CX3CR1^int^ proinflammatory macrophages are recruited to the sites of the damaged intestine to sense bacterial products via TLR and nucleotide-binding oligomerization domain 2, and directly drive the progression of intestinal inflammation by releasing large amounts of proinflammatory mediators such as IL-6, TNF-α, IL-1β, etc. [12, 13]. As a result, Ly6C^hi^CX3CR1^int^ proinflammatory macrophages aggravate the intestinal inflammatory response and play crucial roles in the pathogenesis of CD and UC [14].

Dextran sulfate sodium (DSS)-induced experimental colitis is one of the most widely used models [15]. The DSS colitis model is commonly used in IBD research due to its rapidity, simplicity, reproducibility, and controllability. DSS is delivered by drinking water and causes tissue damage and inflammation, which most closely resembles human UC. In the DSS model, intestinal inflammation results from the impairment of intestinal epithelial cell barrier function by DSS, subsequent exposure of the submucosa to various luminal antigens (bacteria and food) and activation of inflammatory cells involved in innate immunity [16]. Models of acute, chronic and recurrent colitis can be established by changing the concentration and frequency of DSS administration [17]. The DSS-induced colitis model is particularly useful in the study of innate immune-driven colitis, as disease occurs in T and B cell-deficient mice, suggesting that it is driven by innate immune cells [18]. Moreover, studies have suggested that macrophages infiltrating the intestinal mucosa play an important role in maintaining homeostasis by negatively regulating immune responses triggered by commensal bacteria.

Mesencephalic astrocyte-derived neurotrophic factor (MANF) belongs to the fourth family of neurotrophic factors and is an important secreted protein. Although MANF was initially discovered to have neurotrophic activities, further studies revealed that MANF is highly expressed in nonneural tissues and that its cytoprotective activity extends beyond the dopaminergic system [4]. Moreover, MANF is widely expressed in mammalian tissues and is regulated differently by various ER stresses during the course of disease [19, 20]. Previous studies have shown that MANF is expressed in immune cells and has an autocrine immune modulatory function that biases immune cells toward an anti-inflammatory phenotype, thereby promoting tissue repair in both vertebrates and invertebrates and enhancing retinal regenerative therapy [21]. Our group has found that the selective expression of MANF in splenocytes may be involved in plasma cell differentiation and immune regulation [22]. Further study demonstrated that MANF deficiency in macrophages regulates splenic macrophage differentiation in mice [23]. MANF is also defined as a systemic regulator of homeostasis in young animals, especially of liver metabolic homeostasis. A recent study revealed that hepatocyte-derived MANF prevents hepatic steatosis, while immune cell-derived MANF protects against liver inflammation and fibrosis [24]. However, whether and how MANF modulates the function of colonic macrophages during intestinal inflammation remain unknown.

In the current study, we detected MANF expression in inflammatory intestinal tissues of IBD patients, especially in macrophages. We also established DSS-induced colitis in myeloid cell-specific MANF knockout (MKO) mice. We found that MANF deficiency in myeloid cells increased inflammation and disease severity in the colitis mouse model. Moreover, recombinant human MANF (rhMANF) protein administration alleviated the pathological damage of mice with colitis. Interestingly, we found that macrophage-derived MANF reduces the severity of colitis by inhibiting the recruitment of CX3CR1^int^ proinflammatory macrophages and Th17 cells. Mechanistic studies revealed that the protective effect of MANF against colitis is associated with CHOP/BATF2-mediated regulation of inflammation in macrophages.

## Materials and methods

### Human specimens

Clinical specimens were collected from the First Affiliated Hospital of Anhui Medical University (Hefei, China). The use of clinical specimens was in accordance with the Declaration of Helsinki. This study was approved by the Ethics Committee of Anhui Medical University. Basic information concerning the patients, including age and sex, is summarized in Supplementary Table S1. Written informed consent was obtained from all participants.

### MANF knockout mice

C57BL/6J wild-type (WT) mice were purchased from the Model Animal Research Center of Nanjing University (Nanjing, China). MANF^flox/flox^ mice bearing loxP sites flanking exon 3 of the *manf* gene on the C57BL/6 background were kindly provided by Prof. Jia Luo of the University of Kentucky (Lexington, KY, USA). MANF^flox/flox^ mice were cross-bred with LYZ2-cre mice to specifically knock out the *manf* gene in myeloid cells (MKO mice). The mice were born and bred at the same facility and kept on the same rack in an animal housing room that was maintained under specific pathogen–free condition with a controlled temperature (22 °C) and photoperiod (12-h light/12-h dark cycle) and unrestricted access to standard chow and water. All mice were raised in SPF-class environment. All procedures (LLSC20210761) were approved by the Institutional Animal Care and Use Committee of Anhui Medical University.

### Dextran sodium sulfate-induced mouse colitis model

The mice aging 8–10 weeks were administered 3% DSS (MP Biomedical) in drinking water (*w*/*v*) for 7 days. Body weight, rectal bleeding, and diarrhea were scored daily. Clinical colitis scores were calculated according to the following criteria [25]: Rectal bleeding: 0 = no bleeding, 2 = positive hemoccult, and 4 = gross bleeding; diarrhea: 0 = well-formed stools, 2 = soft and pasty stools, and 4 = watery stools. In the survival experiment, the mice were given 4% DSS in the drinking water for 7 days, followed by regular drinking water until the end of the experiment.

For MANF treatment, rhMANF (1 mg/kg) was injected into the tail veins of mice for 3 consecutive days from day 4 to day 6 after DSS challenge, and the same amount of PBS was injected into the tail veins of mice as the controls.

### Cytokine detection by LEGENDplex^TM^ bead-based immunoassay

Cytokines were detected by using a LEGENDplex^TM^ Mouse Inflammation Cytokine Panel array kit (BioLegend, #740165), according to the manufacturer’s guidelines. The serum samples were twice diluted and incubated overnight with TNF-α, IFN-γ, IL-1β, and IL-6 specific beads. Samples were acquired on a BD Celasta Flow Cytometer, and data were analyzed using LEGENDplex™ software.

### RNA extraction and quantitative PCR (qPCR)

RNA was extracted from cells and mouse colon tissues using TRIzol (Invitrogen, #15596018) according to the manufacturer’s instructions. RNA samples were reversely transcribed using PrimeScript^TM^ RT Master MIX (TaKaRa, #RR036A) kits following the manufacturer’s instructions. Quantitative PCR was performed using the SYBR Green Real-time PCR Master Mix (TOYOBO, #QPK-201) kit and the primers were listed in Supplementary Table S2. The samples were run on an ABI QuantStudio5 (Thermo Fisher Scientific). Ct values were normalized to GAPDH, and relative expression level was calculated by the 2^–△△CT^ method.

### Isolation of colonic lamina propria mononuclear cells

The colons were longitudinally opened and cut into 1-cm long pieces. The specimens were washed twice in PBS containing 100 U/mL penicillin and 100 μg/mL streptomycin, and then stirred at 37 °C in 1× Hank’s solution containing 5 mM EDTA and 1 mM dithiothreitol for 30 min to remove colonic epithelial cells. The remaining tissues were cut into pieces and digested with HBSS containing 10% FBS (Gbico, #12657-029), 0.5 mg/mL collagenase IV (Sigma, #C5138), and 5 U/mL DNaseI (Sangon Biotech, #A610099, Shanghai, China), at 37 °C for 30 min. The supernatants were then separated by a 40%–70% Percoll density gradient (GE Healthcare, #17-0891-01), and the cells that layered between the 40% and 70% fractions were collected as lamina propria cells.

### Flow cytometry

The colonic single cells of lamina propria were suspended and stained with mouse monoclonal antibodies, including CD45 (BioLegend, #103132), CD11b (BioLegend, #101206), F4/80 (BioLegend, #123116), CD3 (BioLegend, #100305), B220 (BioLegend, #103223), Ly6C (BioLegend, #128026), CX3CR1 (BioLegend, #149006), SiglecF (BioLegend, #155505), Ly6G (BioLegend, #127613), CD45 (BD, #563891), CD3e (BD, #553061), CD4 (BD, #552051), CD8a (BD, #553030), RORγt (BD, #563081), T-Bet (BD, #563318), and Foxp3 (BD, #560414). The antibodies were incubated at 4 °C in the dark for 30 min. The cell subsets classification was detected by Flow cytometry (BD Celasta). Flow cytometric data were analyzed by using FlowJo software (BD).

### Immunohistochemistry assay

Colon tissues were fixed in 4% formalin, then embedded in paraffin. Sections (4-μm thick) were prepared. The sections were incubated with primary antibodies overnight at 4 °C after blocking with 5% goat serum. The slides were then incubated with secondary antibodies at 37 °C for 1 h according to the manufacturer’s standard protocol (Zhongshan Jinqiao Biotechnology). Then, the sections were stained with 3,3′-diaminobenzidine tetrahydrochloride and hematoxylin. The images were obtained using the microscope (Olympus). The used antibodies include rabbit anti-MANF (Abcam, #ab126321), rabbit anti-BATF2 (Thermo, #PA5-37138), rabbit anti-IL-12 (Bioss, #bs-0767R), rabbit anti-CD68 (Abcam, #ab125212), and rabbit anti-CHOP (Affinity, #AF6722).

The number of positive cells was counted in five randomly selected fields from four sections of each group. The average from five randomly selected fields of each section was used to calculate the mean of each individual for further statistical analysis.

### Immunofluorescence staining

Immunofluorescence assay was performed as previously described [26]. In brief, the paraffin-embedded colonic sections were deparaffinized and rehydrated, and then incubated with 5% BSA for 0.5 h and incubated with antibodies for an additional 2 h at 37 °C. Anti-CD68 and anti-MANF antibodies were used. The nuclei were stained with DAPI for 10 min. The images were taken under the fluorescence microscope (Olympus).

### Histopathology

The distal portion of colon tissues was fixed in 4% paraformaldehyde and then embedded in paraffin. The tissue samples were sectioned at 4-μm thickness and stained with hematoxylin and eosin (H&E) or periodic acid–Schiff (PAS). The severity of colitis was blindly assessed by two pathologists according to the published methods [27]. Colon sections were scored 0–4: 0, normal tissues; 1, mild inflammation in the mucosa with few infiltrating mononuclear cells; 2, moderate amounts of inflammation in the mucosa with a few infiltrating cells, damaged crypt glands and epithelium, mucin depletion from goblet cells; 3, a lot of infiltrating cells in the mucosa and submucosa area, crypt abscesses present with increased mucin depletion and epithelial cell disruption; and 4, massive infiltrating cells in the tissue, complete loss of crypts.

### Western blot

The colon tissues were lysed with RIPA lysis buffer containing protease and phosphatase inhibitor cocktails (Merck, Darmstadt, Germany) and the proteins were detected by Western blot as previously described [28]. The antibodies used include rabbit anti-MANF (Abcam, #ab126321, 1:1000), rabbit anti-CHOP (Santa Cruz, #sc-793, 1:1000), rabbit anti-IL-12 (Bioss, #bs-0767R, 1:1000), rabbit anti-BATF2 (Thermo, #PA5-37138, 1:500), rabbit anti-cleaved caspase-3 (Affinity, #AF7022, 1:1000), and mouse anti-GAPDH (Elabscience, #E-AB-20032, 1:1000). The signal was detected by ECL solution (Beyotime, Shanghai, China, #P0018S), and the images were visualized on ChemiDoc (Bio-Rad, Hercules, CA, USA). The band densities were analyzed by ImageJ.

### Luciferase reporter assays

The pCIneo-MANF-FLAG plasmid was kindly gifted by Prof. Sheng-yun Fang (University of Maryland School of Medicine, USA). Three regions of the BATF2 promoter (−1 to 0 kb, −1.6 to 0 kb, and −2 to 0 kb) were inserted into the Pgl3-basic vector between the *Kpn* I and *Xho* I sites and confirmed by sequencing. The mutant of the BATF2 promoter (−1.6 to 0 kb) that lacks CHOP binding site was purchased from Nanjing Genebay Biotech Co., Ltd.

Luciferase reporter assay was performed according to the manufacturer’s instructions (Dual-Luciferase Reporter Assay System, Promega, #E1910). HEK293T cells were plated in a 24-well plate and transfected with 0.05 μg of cytomegalovirus-Renilla (Promega Corporation, Madison, WI, USA), together with 0.5 μg of BATF2 promoter constructs, with or without FLAG-MANF by using Lipofectamine 2000 transfection reagent (Thermo Fisher Scientific, USA). Twenty-four hours after transfection, the cells were challenged by LPS (100 ng/mL). The lysate was prepared by using Passive Lysis Buffer (Promega) at 24 h after LPS challenge. Luciferase activity was determined from a 20 μL cell extract on a microplate reader. All experiments were performed in triplicate.

### RNA sequencing

The mononuclear cells in colonic lamina propria were isolated and subsequently sorted using PE MACS MicroBeads (Miltenyi Biotech, Bergisch-Gladbach, Germany, #130-048-801). The cells were washed with PBS and labeled with PE-anti-F4/80 antibody, and then the cells were subjected to positive selection using anti-PE magnetic microbeads. Flow cytometry analysis indicated that the cell population contained above 90% F4/80-positive cells. RNA sequencing was performed on the Illumina HiSeq2000 platform by Genewiz (Suzhou, China). The sequence data were deposited in the NCBI Sequence Read Archive under the accession number PRJNA788434.

### TUNEL staining

Cell apoptosis was measured by the YF^®^488 TUNEL Assay Apoptosis Detection Kit (Yuhengbio, T6013) according to the manufacturer’s instructions. TUNEL-positive cells in five randomly selected fields in at least four independent slices were visualized and counted in a blinded manner under a fluorescence microscope (Olympus).

### Statistical analysis

Statistical analysis was performed with GraphPad Prism software. All data are expressed as the mean ± SEM. All experiments were repeated independently at least three times. Statistical significance between two experimental groups was determined using an unpaired, two-tailed Student’s *t* test. Differences with a *P* value of less than 0.05 were considered statistically significant. Three or more groups were compared with ANOVA. Multiple comparisons between variables were assessed by one-way ANOVA with Tukey’s multiple comparisons test. For the mouse survival study, Kaplan–Meier survival curves were generated, and a log-rank test (Mantel–Cox) was used to determine statistical significance.

## Results

### MANF is upregulated in the colon tissues with colitis

**Fig. 1:**
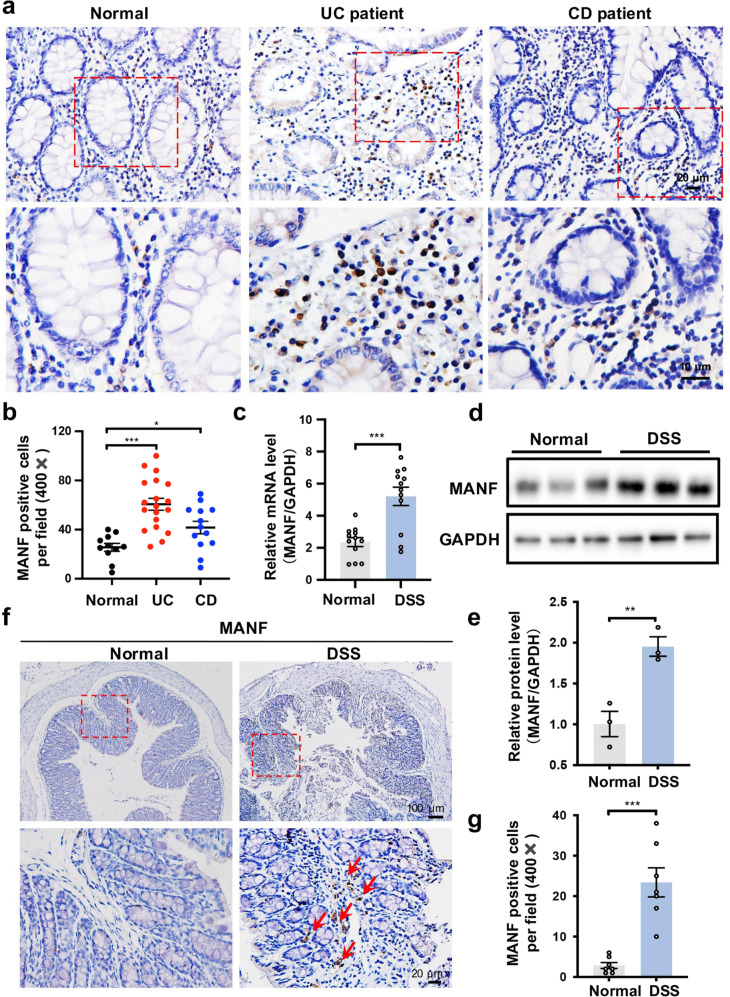
MANF is upregulated in inflammatory colon tissues. **a** Representative immunohistochemical staining of paraffin-embedded sections of colon specimens obtained from patients with UC (*n* = 31), CD (*n* = 13), and healthy controls (*n* = 11). Scale bars: 20 μm (original insets) and 10 μm (enlarged insets). **b** The quantitative date in panel **a**. **c** MANF mRNA levels in colon tissues of control mice and the mice treated with DSS were detected by using qPCR. **d** MANF protein levels in colon tissues of control mice and the mice treated with DSS were detected by Western blot. **e** The quantitative data in panel **d**. **f** MANF protein levels in colon tissues of control mice and the mice treated with DSS were detected by immunohistochemistry assay. **g** The quantitative data in panel **f**. All data are expressed as the mean ± SEM from 6 mice. *P < 0.05, **P < 0.01, ***P < 0.001, compared with normal controls.

To investigate the profile of MANF in IBD, we used immunohistochemistry assay to assess MANF expression in the mucosa of UC and CD patients. Compared with healthy mucosa, MANF expression was significantly increased in the mucosa of UC and CD patients, especially in the mucosa of UC patients (Fig. 1a, b). To further confirm the characteristic of MANF expression in inflammatory intestinal mucosa, we established DSS-induced colitis mouse model and found that MANF was significantly increased both in mRNA and protein levels in mice colon tissues after DSS treatment (Fig. 1c–e). Immunohistochemical staining also revealed that MANF expression was higher in the colon mucosal tissues of the mice treated with DSS than that in the colon mucosal tissues of normal control mice (Fig. 1f, g), which feature is similar to that observed in the mucosa of IBD patients. These results suggest that MANF expression is increased in the inflammatory colon tissues.

### MANF is upregulated in the macrophages with colitis

**Fig. 2:**
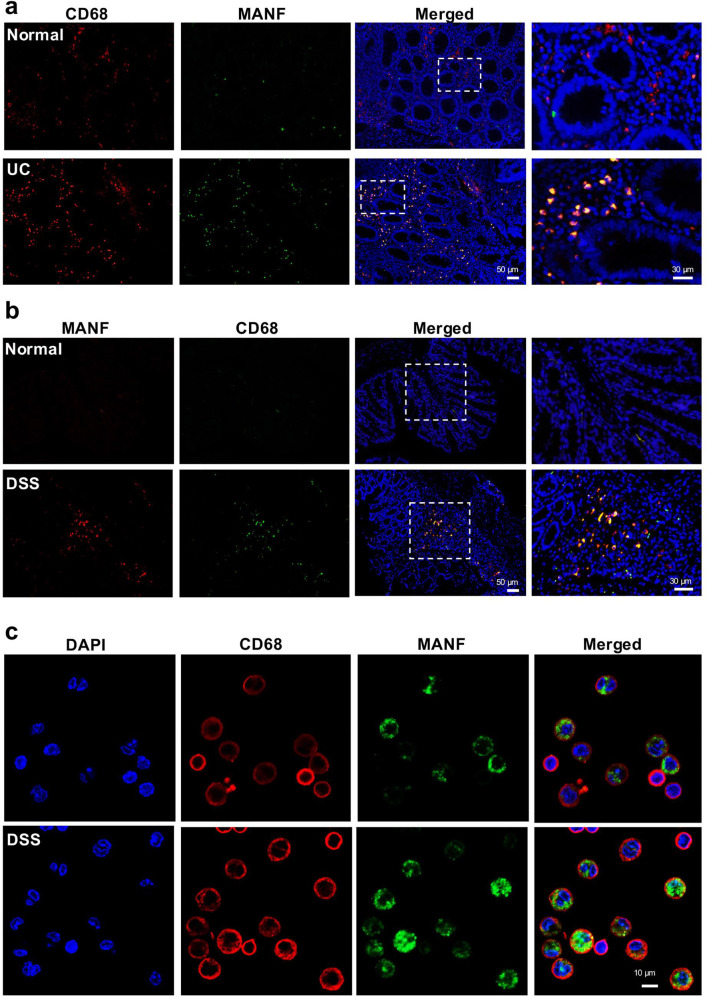
MANF is upregulated in the macrophages of colitis tissues. **a** Immunofluorescence staining for CD68 (red) and MANF (green) in human colon tissues. DAPI (blue) was used to stain nuclei. Scale bars: 50 μm (original insets) and 30 μm (enlarged insets). **b** Immunofluorescence staining for MANF (red) and CD68 (green) in mice colon tissues. DAPI (blue) was used to stain nuclei. Scale bars: 50 μm (original insets) and 30 μm (enlarged insets). **c** Immunofluorescence staining for MANF (green) and CD68 (red) in peritoneal macrophages isolated from mice. DAPI (blue) was used to stain nuclei. Scale bars: 10 μm.

Macrophages are one of the most abundant leukocytes in the colon and play a critical role in colitis by secreting many cytokines and regulating tissue repair [29, 30]. Our previous studies showed that MANF is expressed in macrophages [22]. Considering the significant role of macrophages in IBD, we investigated the expressional characteristic of MANF in the macrophages locating to the colonic mucosa of IBD. At first, we detected the colocalization of MANF and CD68, a marker of activated macrophages in the colonic mucosa of UC patients by using immunofluorescence staining. We found that the number of MANF- and CD68-immunoreactive cells was increased in the colon tissues of UC patients, compared with the normal controls, where MANF and CD68 mostly appear in the same cells (Fig. 2a). To further confirm this result, we also double-labeled MANF and CD68 in colon tissues of mice with colitis. As expected, we also found MANF was upregulated in colonic CD68^+^ macrophages derived from DSS-treated mice, compared with that from normal mice (Fig. 2b). In addition, we detected MANF expression in the peritoneal macrophages of mice with colitis. The result showed that MANF was expressed in some CD68^+^ macrophages and localized in cytoplasm under normal condition. However, MANF was found to appear in the nuclei of some CD68^+^ macrophages after being treated with DSS (Fig. 2c). These results indicate that MANF can be upregulated in inflammatory colon macrophages, which might be involved in IBD progression.

### Myeloid cell-specific MANF knockout aggravates DSS-induced mice colitis

**Fig. 3:**
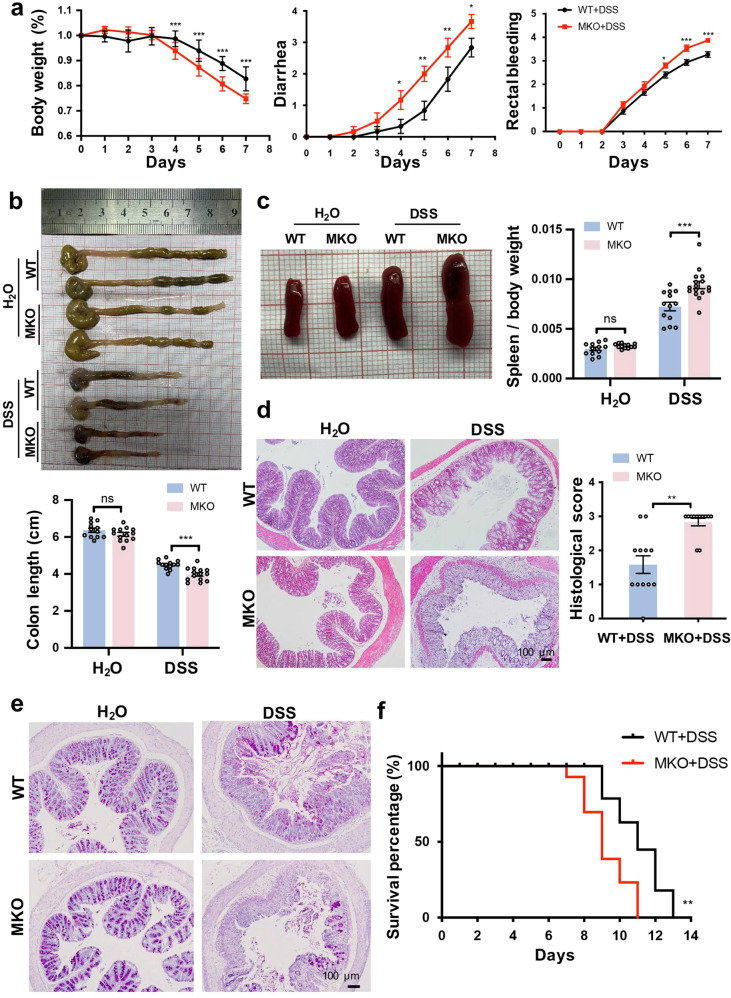
MANF deficiency in macrophages aggravates DSS-induced mouse colitis. **a** The time-dependent curves of body weight, diarrhea, and rectal bleeding. **b** Gross morphology of colons from WT and MKO mice. Colon length was measured on day 8. **c** Gross morphology of spleens from WT and MKO mice. Spleen weight was measured at day 8 and the ratio to body weight was calculated. **d** Representative H&E staining of colon sections and histology scores of WT and MKO mice. Bars: 100 μm. **e** Periodic acid–Schiff staining in colon sections of mice. Bars: 100 μm. **f** Survival percentage of WT and MKO mice. The mice were administered 4% DSS to induce acute colitis and the survival was monitored until day 13. All data are expressed as the mean ± SEM from 12 mice. *P < 0.05, **P < 0.01, ***P < 0.001, compared with WT.

To study the possible role of macrophage-derived MANF in intestinal inflammation, we cross-bred MANF^flox/flox^ mice with Lyz2-Cre mice to generate myeloid cell-specific MKO mice. The efficiency of MKO was validated by PCR (Supplementary Fig. S1a) and Western blotting (Supplementary Fig. S1b). MKO and WT mice were subjected to colitis using 3% DSS. We observed that body weight loss, diarrhea, and rectal bleeding were significantly aggravated in MKO mice, compared with WT mice (Fig. 3a). The colon length of MKO mice was shorter than that of WT mice after DSS treatment (Fig. 3b). Splenomegaly in MKO mice was more severe after DSS treatment (Fig. 3c). Moreover, the infiltrating inflammatory cells were increased, and more severe disruption of the mucosal epithelium in response to DSS treatment were observed in MKO mice, as shown by H&E staining (Fig. 3d). In addition, the epithelium marker E-cadherin protein expression was largely reduced in MKO mice treated with DSS (Supplementary Fig. S1a). Epithelial tight junctions are compromised in GI diseases. Downregulation of occludin and upregulation of the pore-forming tight junction protein claudin-2 lead to altered tight junction structure and pronounced barrier dysfunction [31]. In this study, we also detected occludin and claudin-2, and found that occludin expression was downregulated and claudin-2 expression was upregulated in colonic epithelial cells of DSS-treated WT mice, which is consistent with previous reports. The mRNA level of claudin-2 was significantly higher, while the mRNA level of occludin was significantly lowered in MKO mice than that in WT mice after DSS treatment (Supplementary Fig. S2b, c). These results suggest that MANF deficiency in myeloid cells aggregates the disruption in epithelial barrier integrity. Goblet cells are columnar epithelial cells that secrete mucins. Intestinal mucin is a high molecular weight glycoprotein composed of O-linked glycosides [32]. PAS is used to detect the level of mucin, which reflects the goblet cell population. Accordingly, by PAS staining, we found that more goblet cells lost in MKO colitis mice compared with WT colitis mice (Fig. 3e). The survival ratio was significantly reduced in MKO mice, compared in WT mice after being treated with DSS (Fig. 3f), indicating that myeloid cells-specific MANF deficiency increases the susceptibility of mice to DSS.

Consistently, MKO in myeloid cells significantly increased the number of apoptotic cells in the colon tissues of mice by detecting Annexin V and using TUNEL fluorescence kit assay (Supplementary Fig. S3a, b). The level of cleaved caspase-3 in colon tissues was also significantly increased in MKO mice, compared with WT mice (Supplementary Fig. S3c). These results indicate that MANF deficiency in myeloid cells promotes intestinal epithelium apoptosis.

### Recombinant human MANF (rhMANF) attenuates DSS-induced mice colitis

**Fig. 4:**
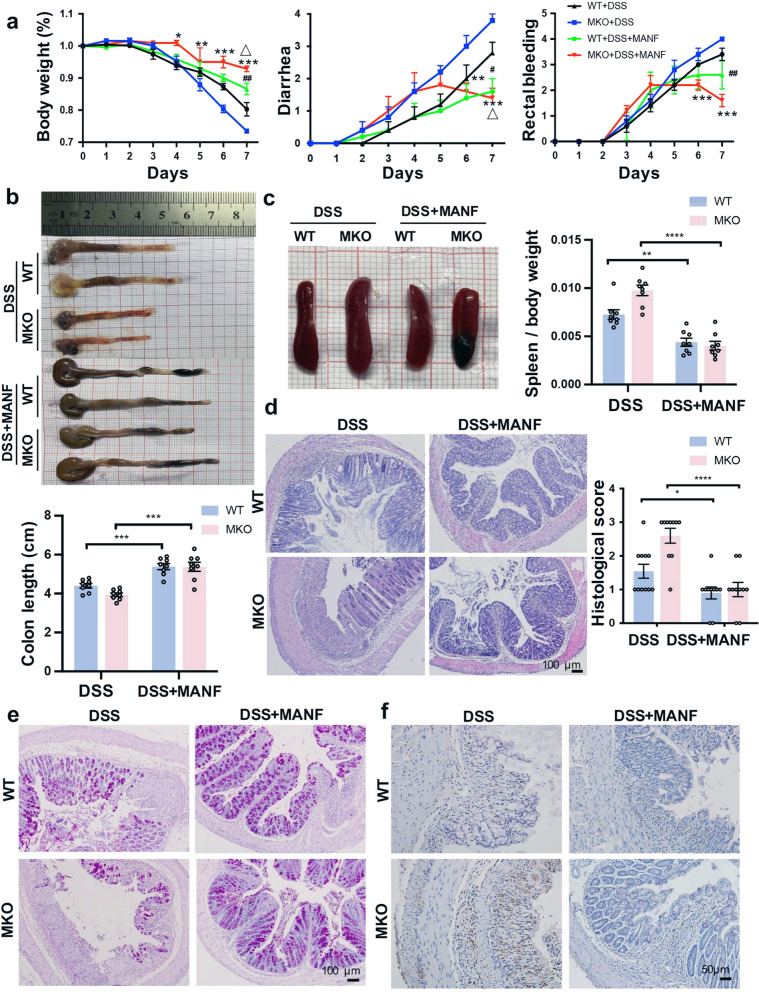
Exogenous rhMANF alleviates DSS-induced mouse colitis. WT and MKO mice were injected by tail vein with His-rhMANF (1 mg/kg) from the 4th day of DSS treatment for 3 consecutive days and then were sacrificed. The control group was injected with PBS in the same way. **a** The time-dependent curves of body weight, diarrhea, and rectal bleeding. Data are collected from three independent experiments with *n* = 10 mice/group in each experiment and expressed as the mean ± SEM. *P < 0.05, **P < 0.01, ***P < 0.001, DSS+MKO vs DSS+MKO+rhMANF; ^#^*P* < 0.05, ^##^*P* < 0.01, DSS+WT vs DSS+WT+rhMANF; ^△^*P* < 0.05, DSS+WT+rhMANF vs DSS+MKO+rhMANF. **b** Gross morphology of colons from WT and MKO mice. Colon length was measured on day 8 (*n* = 10). **c** Gross morphology of spleens from WT and MKO mice. Spleen weight was measured at day 8 (*n* = 10). **d** Representative H&E staining of colon sections and histology scores of WT and MKO mice. Bars, 100 μm. In panels **b** and **c**, data are expressed as the mean ± SEM. *P < 0.05, **P < 0.01, ****P < 0.0001, DSS vs DSS+MANF. **e** Periodic acid–Schiff staining of colon sections. Bars, 100 μm. **f** Immunohistochemical staining of CD68 in colon tissues of WT mice and MKO mice (*n* = 10).

To confirm the effect of MANF on DSS-induced mice colitis, we treated MKO and WT mice with rhMANF by tail vein injection. The schedule of rhMANF treatment is shown in Supplementary Fig. S4a. We firstly verified that His-tagged rhMANF was able to enter colonic cells of mice after injection by tail vein by using immunohistochemistry assay with an antibody against His (Supplementary Fig. S4b). Then we observed the effect of rhMANF on DSS-induced colon injury. We found that rhMANF alleviated colitis symptoms, including body weight loss, diarrhea, and rectal bleeding in WT and MKO mice, and rhMANF was more effective on MKO mice than on WT mice (Fig. 4a). Moreover, rhMANF reduced DSS-induced colon shortening and splenomegaly in WT and MKO mice (Fig. 4b, c). Pathological analyses by H&E staining revealed that rhMANF effectively attenuated the mucosal damage in experimental mice colitis, especially in MKO mice, which was reflected by a significant reduction in the histological score (Fig. 4d). In addition, the loss of goblet cells was alleviated upon rhMANF treatment, which was detected by PAS staining (Fig. 4e). Meanwhile, rhMANF treatment significantly reduced macrophage infiltration in colitis mice, as shown by CD68 staining (Fig. 4f). These results indicated that therapeutic administration of rhMANF attenuates DSS-induced breakdown of the intestinal epithelial barrier and macrophage infiltration in mice colitis.

### Myeloid cell-specific MANF deficiency increases macrophages infiltration in the colon lamina propria

**Fig. 5:**
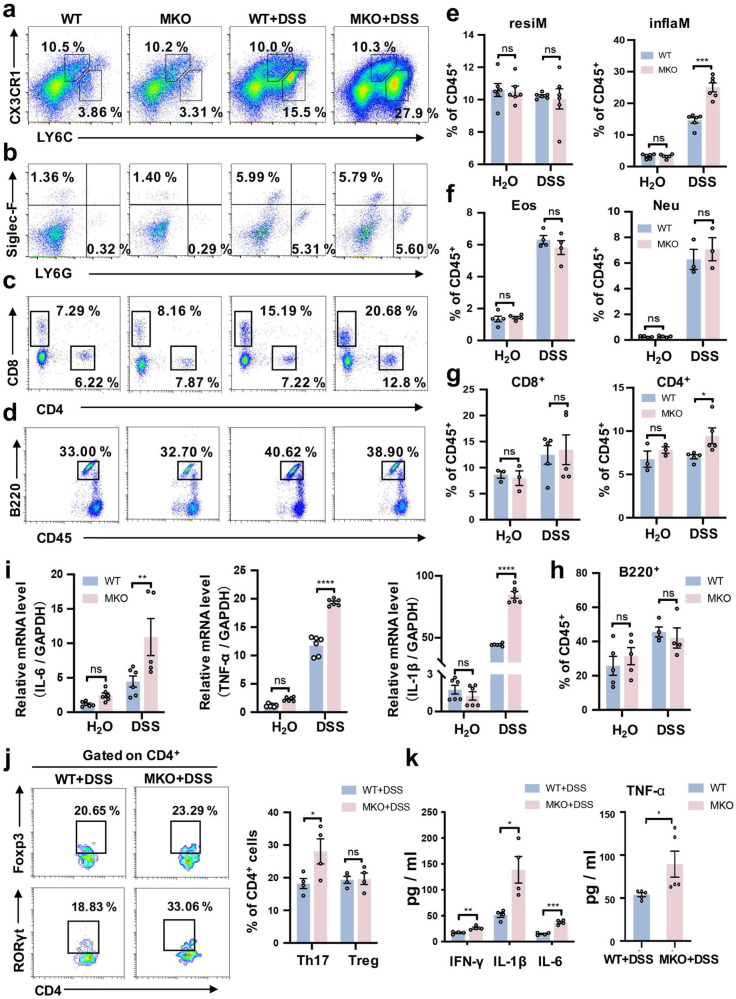
MANF deficiency increases the accumulation of CX3CR1^int^ proinflammatory macrophages. **a**–**d** Representative plots of immune cells (gated on CD45^+^ cells) in colon lamina propria from MKO and WT mice by using flow cytometric analysis. **e** Statistical analysis of macrophages. inflaM: Ly6c^hi^CX3CR1^int^ inflammatory macrophage; resiM: Ly6c^low^CX3CR1^hi^-resident macrophage. **f** Statistical analysis of granulocytes. Neu: neutrophil; Eos: Eosinophils. **g** Statistical analysis of T cells. **h** Statistical analysis of B cells. **i** The mRNA levels of TNF-α, IL-6, and IL-1β in colon tissues were determined by qPCR. **j** Representative plots of T cells (gated on CD4^+^ cells) in colon lamina propria from DSS-treated MKO and WT mice by using flow cytometric analysis. Summary graphs of absolute cell numbers. Treg: CD4^+^Foxp3^+^; Th17: CD4^+^RORγt^+^. **k** The levels of IFN-γ, IL-6, IL-1β and TNF-α in serum were determined by a LEGENDplex^TM^ Mouse Inflammation Cytokine Panel array kit. All data are expressed as mean ± SEM. *n* = 12 mice/group. *P < 0.05, **P < 0.01, ***P < 0.001, MKO vs WT.

To confirm the effect of MANF on macrophage infiltration, we evaluated the impact of myeloid cell-specific MANF deficiency on the subtypes of inflammatory cells in colitis environment. We found that in the water control group, the percentages of immune cells were similar between the two groups of mice. Interestingly, after being challenged with DSS, the percentage of Ly6C^hi^CX3CR1^int^ inflammatory macrophages was significantly higher in the colon lamina propria in MKO mice than that in WT mice, whereas the percentage of Ly6C^low^CX3CR1^hi^-resident macrophages was not different between the WT and MKO mice (Fig. 5a, e). Similarly, the percentage of Ly6G^+^ neutrophils or SiglecF^+^ eosinophils in the colon lamina propria was not different between the WT and MKO colitis mice (Fig. 5b, f). Consistently, the protein levels of inflammatory cytokines TNF-α, IFN-γ, IL-1β, and IL-6 in the serum of MKO colitis mice were increased (Fig. 5k). The mRNA expression of TNF-α, IL-1β, and IL-6 was also significantly increased in the colon tissues of MKO mice with DSS challenge, compared with the WT mice (Fig. 5i). In addition, the expression of the proinflammatory cytokine TNF-α was markedly increased in colon tissues of MKO colitis mice, compared with WT colitis mice (Supplementary Fig. S5a, b). These findings suggest that MANF deficiency in myeloid cells specifically upregulates the subtype of Ly6C^hi^CX3CR1^int^ macrophages, which is closely associated with the local inflammation of colon.

### Myeloid cell-specific MANF deficiency increases the accumulation of Th17 cells in the colon lamina propria

To characterize the impact of MKO on lymphocytes, we detected the subtypes of lymphocytes and found the percentage of CD4^+^ T cells was significantly higher in the colon lamina propria in MKO colitis mice than that in WT colitis mice (Fig. 5c, g). However, the percentage of CD8^+^ T cells in the colon lamina propria was not different between the WT and MKO colitis mice (Fig. 5c, g). Similarly, the percentage of B220^+^ B cells in the colon lamina propria was not different between the WT and MKO colitis mice (Fig. 5d, h). Further analysis of CD4^+^ T cells showed that the increase in the number of CD4^+^ T cells in MKO colitis mice were mainly RORγt-positive Th17 cells (Fig. 5j), which play important roles in the pathogenesis of IBD and experimental colitis [33]. However, there was no significant difference in the number of Foxp3-positive Treg cells between WT and MKO mice after challenge with DSS (Fig. 5j). These results suggest that MANF deficiency in macrophages leads to a significant increase in the accumulation of Th17 cells in the inflammatory microenvironment of colon.

### Myeloid cell-specific MANF deficiency upregulates BATF2 signal in colonic macrophages

**Fig. 6:**
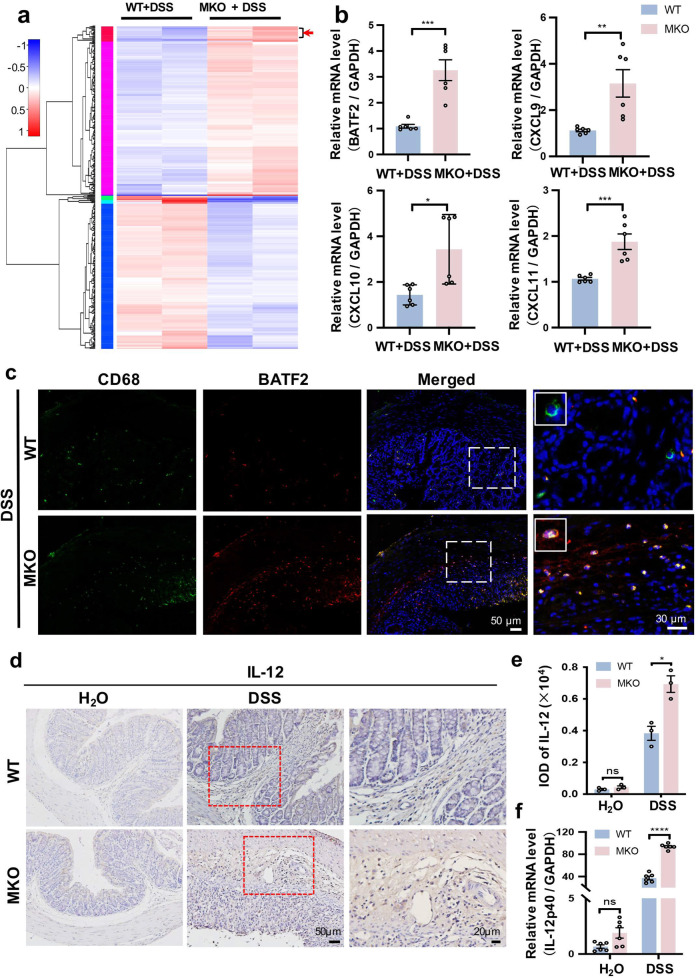
MANF deficiency upregulates BATF2 in colonic macrophages. **a** Heatmaps of genes from RNA-seq analysis of the F4/80^+^ macrophages in colonic lamina propria of DSS-treated WT and MKO mice (*n* = 6). The red indicates upregulated genes, and the blue indicates downregulated genes. **b** The mRNA levels of the indicated genes in colonic macrophages were detected by RT–qPCR assay. **c** Immunofluorescence staining for BATF2 (red) and CD68 (green) in colon tissues of mice treated with DSS. DAPI was stained for nuclei (blue). Bar = 50 or 30 μm. **d** IL-12 was detected by immunohistochemical staining in colon tissues of WT and MKO mice. Bar = 50 or 20 μm. **e** The quantitative data in panel **d**. **f** The mRNA level of IL-12p40 in mice colonic tissues was determined by qPCR. All data are expressed as mean ± SEM. *n* = 6. *P < 0.05, **P < 0.01, ***P < 0.001, ****P < 0.0001, MKO vs WT.

To address the mechanisms involved in the intestinal inflammation triggered by macrophage MANF deficiency, we sorted F4/80^+^ macrophages from colitis mouse colon tissues and performed RNA sequencing. The F4/80^+^ macrophages were typically of >90% purity as determined by flow cytometry (Supplementary Fig. S6a). A cluster analysis of differentially expressed genes from WT and MKO mice was shown in Fig. 6a. We found that MANF deficiency in macrophages upregulated 188 genes and downregulated 170 genes (Supplementary Fig. S6b), and many signaling pathways were affected (Supplementary Fig. S6c). The differentially expressed genes were screened according to their log 2-fold change (log_2_FC) values. Differential gene analysis showed that the transcription factor BATF2 and chemokines CXCL11, CXCL9, and CXCL10 were significantly upregulated in MKO mice (Fig. 6a). Quantitative reverse transcription PCR further confirmed that CXCL9, CXCL10, CXCL11, and BATF2 were highly expressed in colon macrophages knocked out with MANF (Fig. 6b). Immunofluorescence staining also showed that the number of double-labeled macrophages with antibodies against BATF2 and CD68 was increased in the inflammatory colon tissues of MKO mice, compared with that of WT mice (Fig. 6c). Therefore, functional annotation of the differentially expressed genes revealed that MANF deficiency in macrophages led to a markedly increase of various inflammation-related genes, among which BATF2 was the most changed transcription factor. A previous study showed that BATF2 has an antitumor effect through the upregulation of IL-12p40, a component of the bioactive cytokine IL-12, in tumor-associated macrophages [34]. After DSS treatment, the levels of IL-12 and IL-12p40 were markedly upregulated in the colon tissues of MKO mice, compared with that of WT mice (Fig. 6d–f).

### MANF inhibits BATF2 transcription through inhibiting CHOP binding in DSS-induced mice colitis

**Fig. 7:**
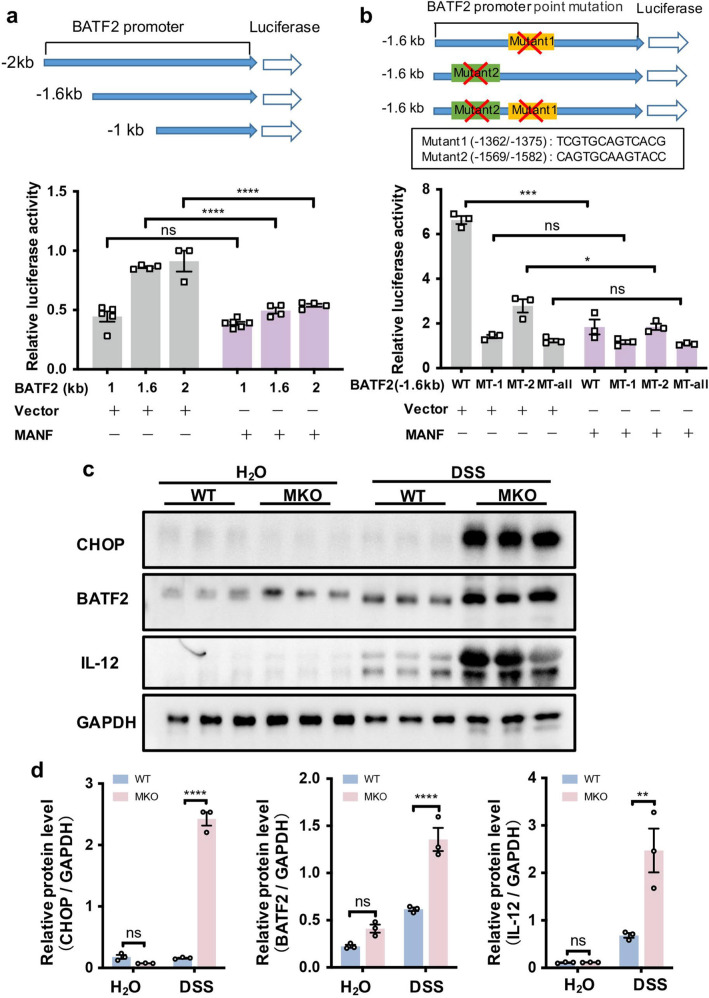
MANF downregulates BATF2 by inhibiting CHOP expression. **a** A schematic diagram of mouse BATF2 promoter and its truncates cloned to the luciferase reporter. The luciferase reporter was co-transfected into 293T cells along or with MANF plasmid. The cells were challenged with LPS at 24 h after transfection, and then the luciferase activity was measured 24 h after LPS stimulation. **b** Effect of mutation of CHOP binding sites on BATF2 transcription. The mutants were co-transfected into 293T cells with MANF plasmid. Twenty-four hours after transfection, the cells were treated with LPS for 24 h, then the luciferase activity was measured. **c** The protein levels of CHOP, BATF2, and IL-12 were detected in colon tissues from the mice treated with DSS. **d** The quantitative data in panel **c**. All data are expressed as mean ± SEM from 3 independent experiments. *P < 0.05, **P < 0.01, ***P < 0.001, ****P < 0.0001.

The above results showed that MKO in macrophages upregulates BATF2 signaling pathway, we wonder how MANF deficiency upregulates BATF2 signaling pathway. Bioinformatics was performed to predict the possible binding sites for transcription factors in the promoter region of BATF2 (−1.6 to −1.0 kb) via TFBIND bioinformatics software (https://tfbind.hgc.jp/) [35]. We found that there are two binding sites of CHOP, a target molecule of MANF [28], in BATF2 promoter region (Fig. 7b). Our previous research found that MANF can inhibit the ATF4-CHOP signaling pathway by regulating the transcription of ATF4 [28]. Consistently, in this study, we found CHOP was dramatically upregulated after MKO (Fig. 7c, d). Therefore, we speculate that MANF may negatively regulate BATF2 transcription via inhibiting CHOP expression. To test this hypothesis, we first performed luciferase reporter assays to examine the regulation of BATF2 by MANF. Three truncates of BATF2 promoter were constructed with a luciferase reporter (Fig. 7a, upper panel). 293T cells were co-transfected with the BATF2 promoter or its truncates and FLAG-MANF. Twenty-four hours after transfection, the cells were treated with LPS for 24 h, and then the luciferase activity was detected. Compared with the vector control, MANF significantly inhibited the transcriptional activity of the BATF2 promoter in the regions of −1.6 and −2 kb, while the region of −1 kb in BATF2 promoter was not affected by MANF (Fig. 7a, lower panel, Supplementary Fig. S7a). Because the region from −1.6 to −1.0 kb in BATF2 promoter has two predicted CHOP binding sites, we next constructed the mutants and deleted the two binding sites (Fig. 7b, upper panel). The results from luciferase reporter gene assay showed that CHOP promoted BATF2 transcription, and CHOP binding site 1 in BATF2 promoter region was a stronger transcription-promoting site (Supplementary Fig. S7b). Consistently, we found that MANF mainly suppressed BATF2 transcription via the binding site 1 of CHOP in the promoter region of BATF2 (Fig. 7b, lower panel). In addition, we observed considerably increased CHOP, BATF2, and IL-12 in MKO colonic tissues, compared with WT controls after DSS challenge (Fig. 7c, d and Supplementary Fig. S7c, d). Therefore, these data suggest that the protective role of MANF in colitis is associated with negatively regulating BATF2 signal-related inflammation via suppressing CHOP expression.

## Discussion

This study uncovers the critical role of MANF in colonic macrophages which contributes to anti-inflammation and tissue repair in colitis. Our data indicate that MANF was significantly upregulated in the colonic mucosal tissues collected from IBD patients and experimental colitis mice, which was highly correlated with pathological colonic damage. We also clarified that the increase of MANF in the colonic tissues was from the infiltrated macrophages, potentially acting as a reactive response to inflammatory stimuli. We further verified that MANF deficiency in myeloid cells exacerbated DSS-induced mice colitis. On the contrary, systemic administration of rhMANF reduced the susceptibility of WT and MKO mice to DSS injury. Most importantly, myeloid cells-specific MKO results in the recruitment of a large number of Ly6C^hi^CX3CR1^int^ proinflammatory macrophages to the colonic mucosal tissues, which leads to a strong proinflammatory response via cytokines and chemokines release that activates Th17 immunity and amplifies intestinal inflammation. These results suggest that upregulation of MANF in the colonic macrophages may have a protective effect on DSS-induced colitis, and adjusting macrophage-derived MANF level may offer a new strategy for IBD therapy.

A lot of previous studies have shown that MANF protects normal cells against the apoptosis induced by various stimuli and promotes the survival and proliferation of normal cells [36–38]. In the present work, we found that the epithelial barrier integrity was severely disrupted by DSS, including loss of goblet cells and massive apoptosis of epithelial cells, in MKO mice. Therefore, we speculate that MANF deficiency-induced damage in epithelial cells may be indirect via the inflammatory environment masked by massive epithelial cell damage caused by DSS chemotoxicity. However, we do not rule out the possible protective mechanisms of MANF in colonic epithelial cells. We found that exogenous rhMANF administered via the tail vein greatly attenuated the severity of colitis in MKO and WT mice. It was reported that radiolabeled MANF does not bind to the cell surface, indicating that it does not have a classical transmembrane receptor [39]. Although His-tagged rhMANF was detected in colon tissues in rhMANF-treated mice by using immunohistochemical staining with anti-His antibody, how the extracellular MANF enters into cells still needs further study.

Epithelial tight junctions are compromised in GI diseases. Inflammation-induced occludin downregulation limits epithelial apoptosis by suppressing caspase-3 expression [40]. Claudins are a family of proteins that are the most important components of tight junctions. In the intestine, claudin-1, −3, −4, −5, and −8 tighten TJ decrease paracellular permeability, whereas claudin-2 forms charge-selective paracellular pores [41]. Downregulation of occludin and upregulation of claudin-2 lead to altered tight junction structure and pronounced barrier dysfunction [31]. Intestinal epithelial claudin-2 expression is upregulated in IBD, where the degree of upregulation correlates directly with disease severity [42, 43]. We found that occludin mRNA was downregulated and claudin-2 mRNA was upregulated in colonic epithelial cells of DSS-treated WT mice, which is consistent with previous reports. The mRNA level of claudin-2 was significantly higher, while the mRNA level of occludin was significantly lower in MKO mice than that in WT mice after DSS treatment. These results suggest that MANF deficiency in myeloid cells aggregates the disruption in epithelial barrier integrity.

In this study, we mainly focused on the impact of MANF on macrophages in colitis. During intestinal inflammation, intestinal macrophages were constantly replenished from the circulating monocytes and the recruited monocytes were dramatically increased. Intestinal monocytes undergo context-dependent phenotypic and functional adaptations to either maintain local immune balance or support intestinal inflammation [44]. Ly6C^hi^ monocytes can differentiate all the subtypes of macrophages, including CX3CR1^hi^-resident macrophages and CX3CR1^int^ inflammatory macrophages in colon. CX3CR1^int^ pool represents a continuum in which newly recruited, recently differentiated monocytes develop into CX3CR1^hi^-resident macrophages. In experimental colitis, the differentiation of the cells in CX3CR1^int^ pool was arrested and the TLR-responsive CX3CR1^int^ proinflammatory macrophages were accumulated [14]. Resident and inflammatory macrophages in the colon represent alternative differentiation outcomes of the same precursor. Disruption of the differentiation of Ly6C^hi^ monocytes into resident macrophages can induce inflammation. A constant balance between resident macrophages and inflammatory macrophages is essential for maintaining gut homeostasis and ensuring protective immunity when required [45]. However, the key factor involved in this kind of balance remains unclear. Here, we found that myeloid cell-specific MKO significantly increased the proportion and number of Ly6C^hi^CX3CR1^int^ proinflammatory macrophages, while the proportion and number of resident macrophages remained basically unchanged. Meanwhile, the expression of the proinflammatory cytokines IL-6, IL-1β, TNF-α, and IL-12p40 was significantly increased in the inflammatory intestinal tissues in MKO mice. These results suggest that endogenous MANF of myeloid cells plays an important role in maintaining the balance between the circulating inflammatory macrophages and resident macrophages.

Previous studies have shown that the activated monocytes/macrophages can affect the Th1/Th17 cells differentiation from CD4^+^ T cells by secreting cytokines and chemokines [46]. We further found that myeloid cell-specific MANF knockdown also promoted the recruitment of Th17 cells to colon tissue. Th17 cells are highly associated with the pathogenesis of IBD [47]. Under physiological conditions, Th17 cells regulate the integrity of the physical barrier of epithelial cells through the chemotaxis of neutrophils and macrophages, and stimulate the epithelial cells to produce antimicrobial peptides, which play an important role in the intestinal mucosal barrier [48]. However, under pathological conditions, a large number of Th17 cells infiltrate into the inflammatory GI mucosa of IBD patients and aggravate the progression of the disease via secreting proinflammatory mediators [49, 50]. These findings suggest that macrophage-derived MANF may play an immunomodulatory role in colitis via driving adaptive Th17 immunity.

Proinflammatory cytokines and chemokines play an important role in the pathogenesis of colitis [51]. To clarify the mechanisms of how MANF regulates macrophages, we analyzed the differential genes expressed in the colonic macrophages between WT and MKO colitis mice by RNA sequencing assay. We found that CXCL9, CXCL10, and CXCL11 were significantly increased in MANF-deficient macrophages, which is consistent with a previous report that found CXCR3 axis was significantly upregulated in inflammatory colon tissues of children with CD and UC [52]. The expression of specific chemokines is strongly correlated with the degree of local inflammation and tissue damage in patients with IBD. Studies have shown that CXCL9, CXCI 10, and CXCL11 are involved in the immune dysfunction of target organs and over-amplification of local inflammatory responses at lesion sites in many autoimmune diseases, including IBD [53, 54].

BATF2 belongs to the basic leucine zipper transcription factor family. The BATF family was initially characterized as the inhibitors of tumor growth through the suppression of AP-1 activity [55]. BATF2 is predominantly expressed in monocytes/macrophages [56]. Recent studies have shown that BATF2 is important for appropriate innate immune responses. Roy et al. found that BATF2 knockdown with shRNA resulted in the downregulation of several important immune-regulatory genes, including Nos2, TNF-α, CCL5, CXCL9, CXCL11, CCR5, CXCR3, IL-6, and Niarc1 in IFN-γ or LPS-activated macrophages [57]. In our RNA-seq results, we found that MANF deficiency in myeloid cells upregulated 188 genes and downregulated 170 genes with DSS challenge, BATF2 was the most upregulated transcription factor, which is consistent with the findings of Roy et al. that BATF2 was significantly induced in IFN-γ or LPS-activated classical macrophages, while there was almost no effect in alternatively activated or unstimulated macrophages [57]. DSS is able to cause severe disruption in the colonic epithelial monolayer, which resulted in the rapid spread of intestinal Gram-positive or Gram-negative bacteria to the submucosa and rapidly stimulated immune cells in the lamina propria of the intestine and led to an acute inflammatory response [17]. Moreover, BATF2 was also significantly induced during mycobacterium tuberculosis (Mtb) infection with classically activated macrophages. It was found that shRNA-mediated BATF2 knockdown in heat-killed Mtb-stimulated macrophages resulted in a decrease in expressing Nos2, TNF-α, CCL5, and IL-12B [56]. Consistently, a study also identified that the expression of proinflammatory chemokines (CXCL2, CXCL3), proinflammatory cytokines (IL-1α, IL-12), and killing effector molecules (Nos2) was significantly reduced in BATF2-deficient macrophages compared with the macrophages from WT mice [58]. In addition, in tumor-associated macrophages, the expression of IL-12p40 was facilitated through the interactions between BATF2 and the p65/p50 heterodimer, which induces the antitumor adaptive immune responses of CD8^+^ T cells [34]. Therefore, MANF negatively regulates the expression of BATF2-mediated proinflammatory cytokines and chemokines in colonic macrophages, which may account for its role in controlling colitis.

In our study, MANF deficiency in macrophages also led to an increase in BATF2, which caused a significant upregulation of IL-12p40 as a result. Genome-wide association studies and several case–control association studies have confirmed IL-12B gene as a susceptibility locus of both CD and UC across different racial and ethnic groups [59, 60]. IL-12p40 is a subunit shared by IL-12 and IL-23, which is encoded by IL-12B gene. IL-12p40 is an important cytokine in the IL-12/23 pathway and plays a crucial role in driving intestinal inflammation. Some studies demonstrated that monoclonal antibodies against IL-12p40 abrogated experimental colitis in mice [61, 62]. Ustekinumab, a fully human IgG1κ IL-12p40 monoclonal antibody, is effective in the induction and maintenance therapy of refractory CD [63]. In addition, studies have revealed that IL-12p40 is upregulated in IBD patients, and IL-12B mRNA expression has a significantly positive correlation with T-bet or RORγt mRNA expression at both systematic and local levels, suggesting that IL-12p40 might be tightly associated with Th1 or Th17 immune responses [64].

To search the regulators in BAFT2 transcription, we analyzed the promoter region of BATF2 by using TFBIND bioinformatics software (https://tfbind.hgc.jp/) and found two potential binding sites of CHOP. CHOP is a transcription factor, minimally expressed in the cytoplasm under normal condition, but upregulated in the nucleus under stress [65]. Our previous data showed that MANF can inhibit CHOP expression by negatively regulating ATF4-CHOP signaling pathway via binding to ATF4 promoter [28]. Consistently, we found that in colitis, MANF deficiency in myeloid cells significantly upregulated CHOP expression. We also clarified that CHOP promotes BATF2 transcription and predominantly binds to the binding site 1. As expected, MANF significantly inhibited the transcriptional activity of BATF2. Collectively, our data indicate that upregulation of CHOP caused by myeloid MKO promotes BATF2 transcription, which leads to the activation of BATF2-mediated inflammatory responses and aggravates intestinal inflammation. In another word, MANF functions as a negative regulator in macrophages to downregulate CHOP-BATF2 signaling pathway, thereby attenuated colitis.

## Conclusion

**Fig. 8:**
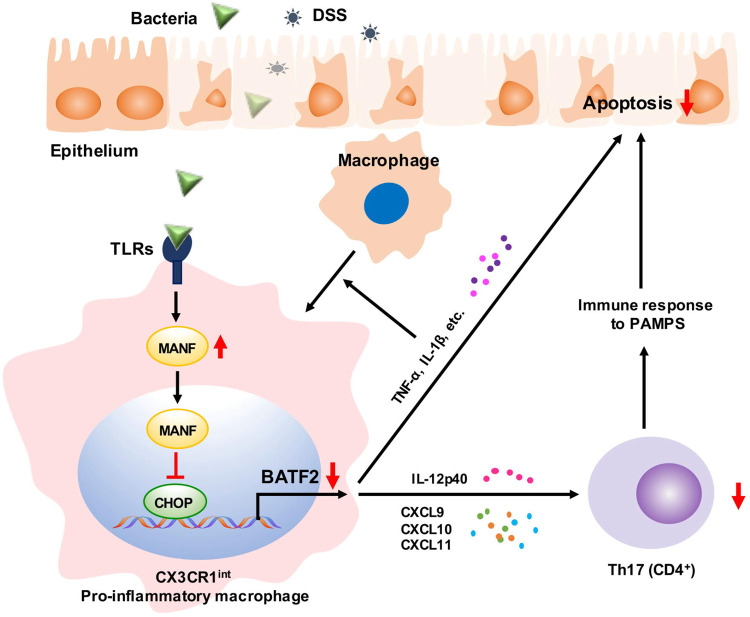
Schematic diagram of MANF ameliorating colitis. The macrophages in colonic tissue are activated by invading microbes or DSS and produce cytokines, which stimulate macrophages to differentiate to CX3CR1^int^ proinflammatory macrophages. On the other hand, MANF is upregulated in macrophages and transferred to the nuclei, where it inhibits CHOP expression during this process. Because CHOP positively regulates BATF2 transcription by binding to BATF2 promoter, the expression of BATF2 downstream genes, including the inflammatory chemokines (CXCL9, CXCL10, CXCL11) and proinflammatory cytokines (TNF-α, IL-1β, IL-12p40) was enhanced, which results in Th17 cells activation and intestinal epithelial cells apoptosis. Consequently, MANF ameliorates colitis and repairs intestinal injury via inhibiting CHOP-BATF2 signaling.

In summary, our study demonstrates that MANF restricts the recruitment of Ly6C^hi^CX3CR1^int^ proinflammatory macrophages to colon tissue and promotes tissue repair in DSS-induced colitis by inhibiting CHOP-BATF2 signaling pathway via BATF2-mediated innate immune pathway and Th17-dominated adaptive immune responses, respectively (Fig. 8).

## Supplementary information

Supplementary Information
