# Supplementary material for: MANF ameliorates DSS-induced mouse colitis via restricting Ly6ChiCX3CR1int macrophage transformation and suppressing CHOP-BATF2 signaling pathway
Source: Acta Pharmacol Sin. 2023 Jan 12;44(6):1175–90. doi: 10.1038/s41401-022-01045-8 (PMC10202914; doi:10.1038/s41401-022-01045-8)
Supplement: Supplementary file 1 — Supplementary Information [file 41401_2022_1045_MOESM1_ESM.docx]

**Supplementary Fig. S1**

**Supplementary Fig. S1. Identifying the efficiency of myeloid cell-specific MANF knockout.**

(a) Identification of MANF knockout in myeloid cells of mice by PCR assay. Primer P5 (P5) was used to detect the insertion of Lyz2-Cre enzyme sequence. A band of 750 bp was amplified in MKO mice by primer P5. Primer P2 (P2) was used to detect the insertion of LoxP sites. A band of 513 bp was amplified in MKO mice and 306 bp in WT mice by P2 primers. (b) Identification of MANF knockout in myeloid cells of mice by Western blot assay.

**Supplementary Fig. S2**

**Supplementary Fig. S2.** **MANF deficiency in myeloid cells aggravates the destruction of epithelial integrity in mice colitis.**

(a) Immunofluorescence staining for E-cadherin (red) and DAPI for nuclei (blue) in colon tissues from the mice treated with DSS. Scale Bar=50 μm. (b) The mRNA levels of claudin-2 and occludin in mice colonic epithelial cells were determined by qPCR. Data are expressed as mean ± SEM. *n* = 9. **P* < 0.05, MKO vs WT.

**Supplementary Fig. S3**

**Supplementary Fig. S3. MANF deficiency in myeloid cells induces apoptosis in intestinal epithelial cells.**

(a) Immunofluorescence staining for Annexin V (red) and DAPI for nuclei (blue) in colon tissues from the mice treated with DSS. Scale bar=50 μm. (b) TUNEL staining was used to evaluate apoptotic cells in colon tissues. Scale bar=50 μm. (c) Cleaved caspase-3 level was detected by immunoblot in colon tissues from the mice treated with DSS. The relative level of cleaved caspase-3 was normalized by GAPDH. Data are expressed as mean±SEM. *n*=9. ****P* < 0.001, MKO vs WT.

**Supplementary Fig. S4**

**Supplementary Fig. S4.** **Schedule for** **rhMANF injection to colitis mice.**

(a) Time schedule for rhMANF administration. (b) Detection of His-MANF in colon tissues after administration of rhMANF via tail vein by using immunohistochemical staining with antibody against His. The scale bars are shown as indicated.

**Supplementary Fig. S5**

**Supplementary Fig. S5. MANF knockout in macrophages promotes TNF-α secretion.**

(a) TNF-α level in colon tissues of mice treated with DSS was detected by immunohistochemical staining. The scale bars are shown as indicated. (b) The quantitative data in panel a. Data are expressed as mean ± SEM. *n* = 6. ****P* < 0.001, MKO vs WT.

**Supplementary Fig. S6
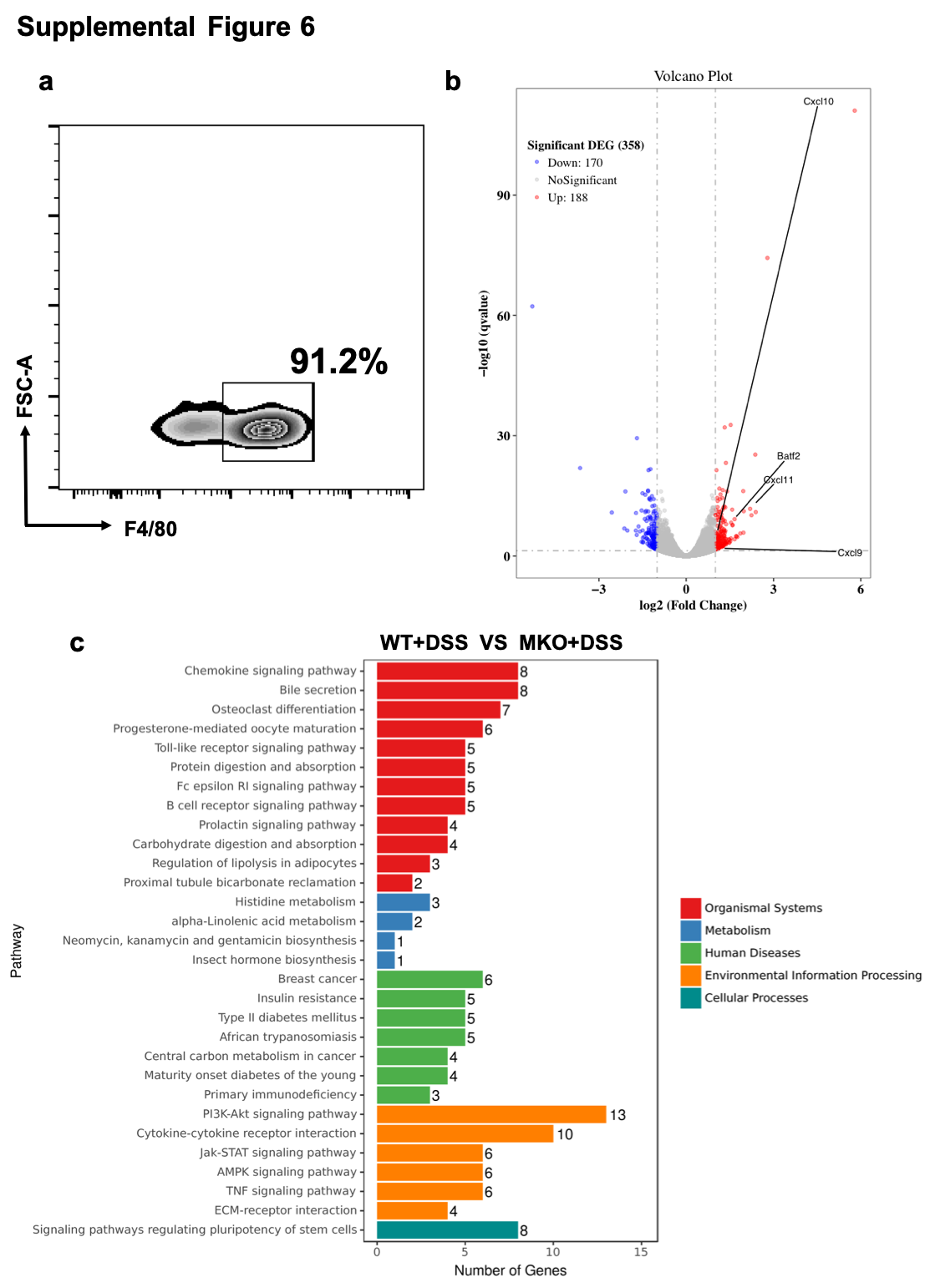
**

**Supplementary Fig. S6. Differential genes in F4/80^+^ macrophages of colonic lamina propria between WT and MKO mice.**

(a) The F4/80^+^ macrophages in colonic lamina propria of mice was sorted by flow cytometry with magnetic beads. (b) Volcano plot of the differentially expressed genes between WT and MKO mice with DSS-induced colitis. The red indicates upregulated genes, and the blue indicates downregulated genes. (c) KEGG enrichment analysis of the top 20 differentially expressed genes in the top 20 pathways after myeloid cell -specific MANF knockout.

**Supplementary Fig. S7**

**Supplementary Fig. S7. MANF deficiency in macrophages upregulated the expression of CHOP and BATF2.**

(a) The Relative luciferase activity of BATF2 promoter and its truncates. (b) Effect of CHOP on BATF2 promoter. BATF2 promoter and its mutants were co-transfected into 293T cells with CHOP plasmid. Twenty-four hours after transfection, the cells were activated with LPS for 24 h. Then, the luciferase activity was measured. (c) BATF2 level in colon tissues from the mice treated with DSS was detected by immunohistochemical staining. Scale bars: 100 μm (original insets) and 50 μm (enlarged insets). (d) Immunohistochemical staining of CHOP level in colon tissues from the mice treated with DSS was detected by immunohistochemical staining. Scale bars: 100 μm (original insets) and 50 μm (enlarged insets). All data are expressed as mean ± SEM. *n* = 12. **P* < 0.05, ****P* < 0.001, compared as the indicated.

**Supplementary Table S1. Basic information of normal control, UC and CD patients from the First Affiliated Hospital of Anhui Medical University.**

|  | **Control** | **CD** | **UC** |
| --- | --- | --- | --- |
|  | n=11 | n=13 | n=31 |
| **Age(years)** |  |  |  |
| **≤30** | 3 | 8 | 11 |
| **30-50** | 3 | 3 | 15 |
| **≥50** | 5 | 2 | 5 |
| **Gender** |  |  |  |
| **Male** | 6 | 6 | 13 |
| **Female** | 5 | 7 | 18 |

**Supplementary Table S2. Primers for qPCR analysis.**

| **Genes** | **Forward primer (5’-3’)** | **Reverse primer (5’-3’)** |
| --- | --- | --- |
| GAPDH | CAACTTTGGCATTGTGGAAGG | ACACTTTGGGGGTAGGAACAC |
| TNF-α | CCTGTAGCCCACGTCGTAG | GGGAGTAGACAAGGTACAACCC |
| IL-6 | ACACATGTTCTCTGGGAAATCGT | AAGTGCATCATCGTTGTTCATACA |
| IL-1β | ATGGCAACTGTTCCTGAACTCAACT | CAGGACAGGTATAGATTCTTTCCTTT |
| CXCL11 | CCGAGTAACGGCTGCGACAAAG | CCTGCATTATGAGGCGAGCTTG |
| CXCL9 | AATGCACGATGCTCCTGCA | AGGTCTTTGAGGGATTTGTAGTGG |
| IL-12p40 | CAGAAGCTAACCATCTCCTGGTTTG | TCCGGAGTAATTTGGTGCTTCACAC |
| claudin-2 | CAACTGGTGGGCTACATCCTA | CCCTTGGAAAAGCCAACCG |
| occludin | TTGAAAGTCCACCTCCTTACAGA | CCGGATAAAAAGAGTACGCTGG |
| BATF2 | GCCCAGCGC AGCCGGCAGAA | CCAGCTCAGTCTGCAAGGCCT |
